# The data on metagenomic profile of bacterial diversity changes in the different concentration of fermented romaine lettuce brine

**DOI:** 10.1016/j.dib.2019.104190

**Published:** 2019-06-25

**Authors:** Trina Ekawati Tallei, Fatimawali, Johanis Jullian Pelealu

**Affiliations:** a Department of Biology, Faculty of Mathematics and Natural Sciences, Sam Ratulangi University, Indonesia; b Pharmacy Study Program, Faculty of Mathematics and Natural Sciences, Sam Ratulangi University, Indonesia

**Keywords:** Fermentation, Metagenome, Illumina, Lettuce, Diversity

## Abstract

This article describes the data on metagenomic profile of the bacterial community and diversity in the brine of fermented romaine lettuce in two experimental brine salinity (5 and 10%) obtained by Illumina MiSeq sequencing of V3–V4 region of 16S rRNA gene. A total of 98,660 reads (10%) and 95,968 (5%) consisted 38 and 47 consensus lineages (OTUs), respectively. Predominating phyla in 10% were 55.89% Proteobacteria and 41.97% Firmicutes, while predominating phyla in 5% brine were 63.47% Proteobacteria and 34.60% Firmicutes. The predominating lower taxa in 10% brine were Haererehalobacter salaria (54.19%) and Bacillaceae (33.2%), while in 5% brine were Enterobacteriaceae (46.67%) and Bacillus (21.53%). Leuconostoc (6.97%) and Lactococcus (3.97%) only appeared in 5% brine. The data presented here is the first metagenomic profile of romaine lettuce fermentation in different brine concentration and will serve as a baseline to understand the shifting of bacterial diversity associated with the change in brine concentration.

**Table:** Specifications table

Subject area	*Biology*
More specific subject area	*Microbiome*
Type of data	*Table, graph, figure*
How data was acquired	*Microbiome metagenomic profiling obtained by Illumina MiSeq sequencing of V3–V4 region of 16S rRNA genes*
Data format	*Raw, filtered, and analysed*
Experimental factors	*Romaine lettuces were fermented anaerobically in 5% and 10% brine for 4 days at room temperature in the dark. The brine was served as collection of microbiome. Extracted bacterial genomic DNAs were used as templates to amplify V3–V4 region of the 16S rRNA genes. An Illumina two-step PCR protocol was used to prepare the amplicon library. The paired-end sequences were generated in 2x300bp format from MiSeq.*
Experimental features	*The filtered sequence reads were analysed using bioinformatics pipeline.*
Data source location	*Department of Biology, Faculty of Mathematics and Natural Sciences, Sam Ratulangi University, Manado – North Sulawesi - Indonesia*
Data accessibility	*Data are included in this article.*

**Table:** 

**Value of the data** • This is the first metagenomic profiling report on Romaine lettuce fermentation in different brine concentration. • The data gives insight on the shifting of bacterial community in different concentration of brine in Romaine lettuce fermentation. • The data can be regarded as base line to the safe Romaine lettuce fermentation.

## 1 Data

**Fig. 1:**
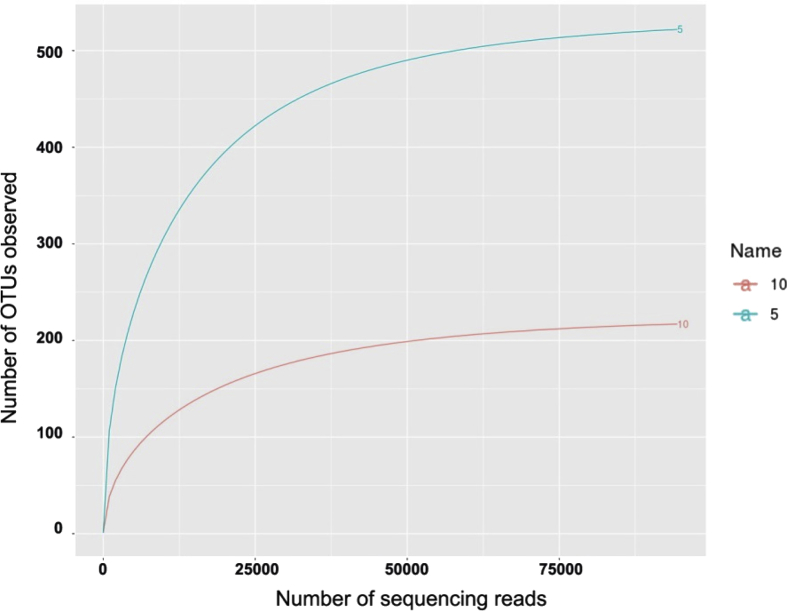
Observed rarefaction curves that estimate the OTU richness in the brine of Fermented Romaine lettuce obtained from the Illumina Hiseq raw data. The OTU diversity for each sample was obtained using V3–V4 primers of 16S rRNA.

This dataset describes the effect of different concentration of brine in the fermentation of lettuce to evaluate the shifting of the bacterial community during the fermentation process using metagenomic data. Salt is mainly used in fermentation as preservative because it has a capability to reduce the water activity of foods [1] therefore the type and extent of microbial activities are inhibited. The data presented here was obtained by Illumina MiSeq sequencing of V3–V4 regions of 16S rRNA gene. Rarefaction plots (Fig. 1) revealed that the sequencing coverage were sufficient for data comparison, as all samples entered the plateau phase. The observed OTUs sequences in the curve has not been combined together according to their respective taxonomical assignations, therefor the numbers of OTUs observed are higher than presented in Table 1 (see section 2.3 for the procedure).

**Table 1:** The identified phyla from 10% to 5% brine of fermented Romaine lettuce.

Phyla	10%			5%		
	OTU	Reads	Percentage	OTU	Reads	Percentage
Proteobacteria	13	55140	55.89%	17	60909	63.47%
Firmicutes	15	41408	41.97%	21	33209	34.60%
Cyanobacteria	1	1381	1.40%	1	308	0.32%
Actinobacteria	7	101	0.10%	5	1021	1.06%
Planctomycetes	1	47	0,05%	2	11	0.01%
Armatimonadetes	1	2	0.002%	1	5	0.005%
Unassigned		581	0.59%		505	0.53%

**Fig. 2:**
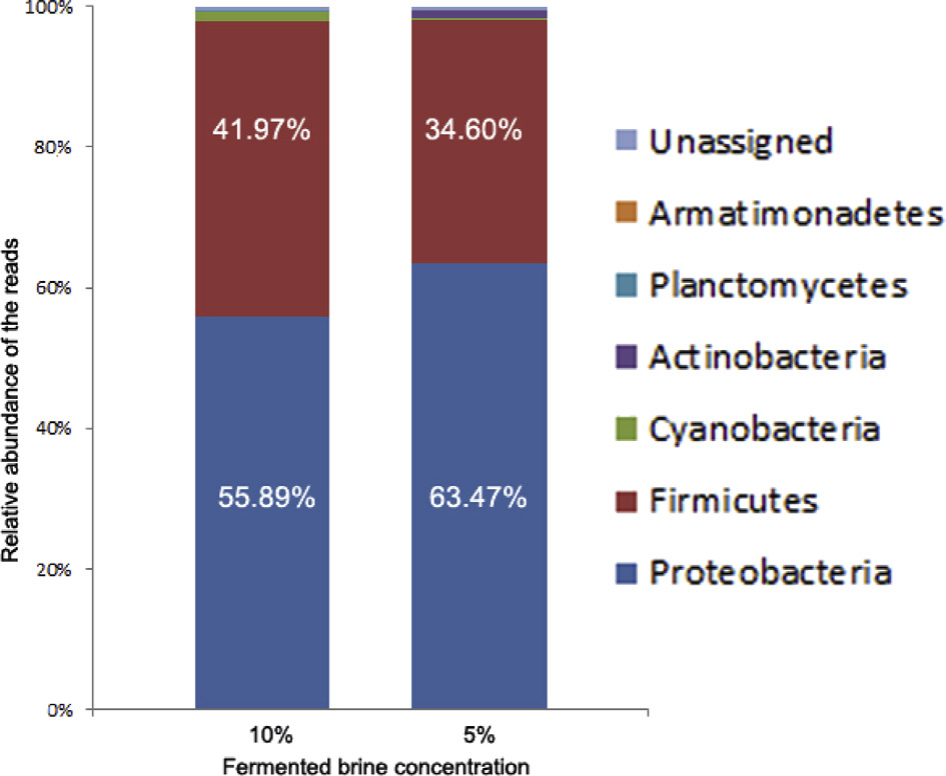
Relative abundance of reads in 10% and 5% brine of fermented lettuce obtained by NGS.

After removal of chimera and singleton, the OTUs were clustered according to 97% degree of similarity. The OTUs which belong to the same taxa were then combined together. A total of 6 phyla and 1 unassigned were generated. A total of 38 OTUs from 98,660 reads and 47 OTUs from 95,968 reads was found in 10% and 5% of fermented brine of Romaine lettuce, respectively. As shown in Fig. 2 and Table 1, most bacteria in the 10% fermented brine belonged to Proteobacteria (13 OTUs, 55,140 reads, 55.89% of the 98,660 reads in total) and Firmicutes (15 OTUs, 41408 reads, 41.97% of the total reads). The minority belonged to Cyanobacteria (1 OTU, 1.381 reads, 1.4% of the total reads), Actinobacteria (7 OTUS, 101 reads, 0.10% of the total reads), Planctomycetes (1 OTU, 47 reads, 0.05% or the total reads), Armatimonadetes (1 OTU, 2 reads, 0.002% of the total reads, and unassigned (581 reads, 0.59% of the total reads).

Most bacteria in the 5% fermented brine belonged to Proteobacteria (17 OTUs, 60,909 reads, 63.47% of the 95,968 reads in total) and Firmicutes (21 OTUs, 33,209, 34.60% of the total reads). The minority belonged to Actinobacteria (5 OTUs, 1,021 reads, 1.06% of the total reads), Cyanobacteria (1 OTU, 308 reads, 0.323% of the total reads), Planctomycetes (2 OTUs, 11 reads, 0.01% of the total reads), Armatimonadetes (1 OTU, 5 reads, 0.01% of the total reads), and unassigned (505 reads, 0.53% of the total reads). Cyanobacteria are naturally found in all types of water [2]. The data shows the shift of the bacterial community structure in the 5% and 10% brine.

**Fig. 3:**
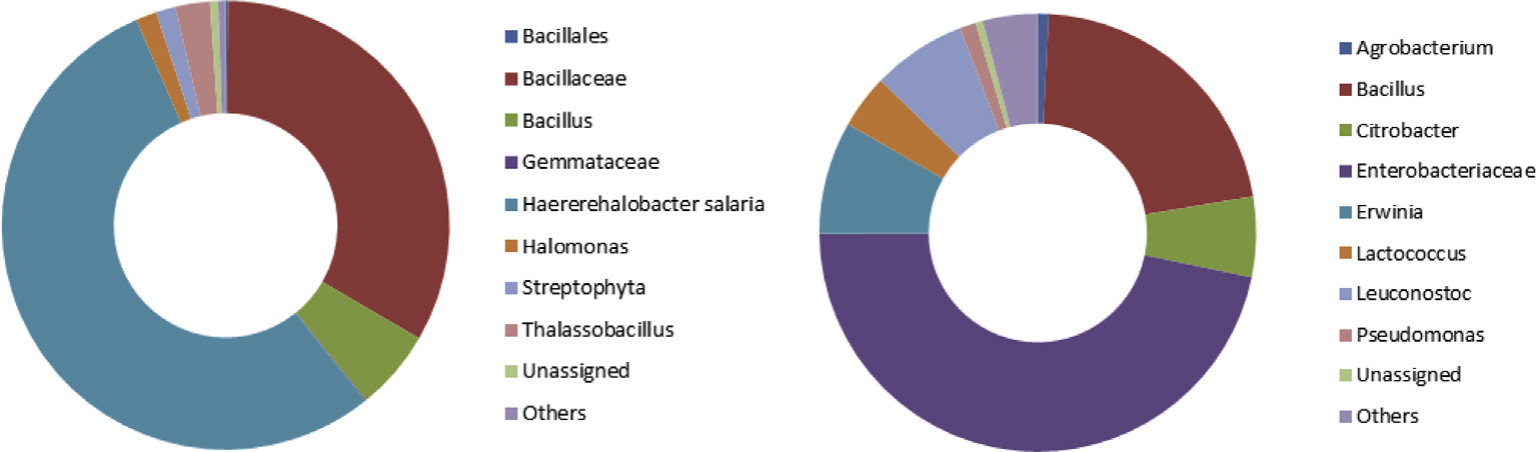
Taxonomic classification of OTUs at lower taxa level for sample of concentration 10% (left) and 5% (right) of fermented Romaine lettuce brine. Only the top 10 lower taxa are summarized here.

The top 10 lower taxa including unassigned OTUs are depicted in Fig. 3. The common dominating core genus in both samples was *Bacillus*. There were also some common lower taxa in both samples, however the amount was very unbalanced. One was found very high in 5% brine, but very low in 10% brine, and vice versa. The data showed a shift in the lower taxa of the bacterial community in both samples. The predominating lower taxa in 10% brine included *Haererehalobacter salaria* (p:Proteobacteria; c:Gammaproteobacteria; o:Oceanospirillales; f:Halomonadaceae) (54.19% of the total reads), Bacillaceae (p:Firmicutes; c:Bacilli; o:Bacillales) (33.20% of the total reads), and *Bacillus* (5.8% of the total reads). In 5% brine, the predominating lower taxa included Enterobacteriaceae (p:Proteobacteria; c:Gammaproteobacteria; o:Enterobacteriales) (46.67% of the total reads), *Bacillus* (51.53% of the total reads), *Erwinia* (8.32% of the total reads), *Leuconostoc* (6.98% of the total reads), *Citrobacter* (6.92% of the total reads), and *Lactococcus* (3.97% of the total reads).

*Agrobacterium*, *Erwinia*, *Exiguobacterium*, *Lactococcus*, *Leuconostoc*, and *Pseudomonas* were not detected in the 10% brine. There are some insignificant reads of *Bacillus flexus*, *Citrobacter*, *Klebsiella*, and Micrococcaceae in 10% brine, however they can be found abundantly and quite abundantly in 5% brine. *Haererehalobacter salaria* and other Bacillaceae were the most abundant in 10% brine, while Enterobacteriaceae and *Bacillus* were the most abundant in 5% brine. Alpha diversity indices are shown in Table 2. The p-values corresponding to the F-statistic of one-way ANOVA were lower than 0.05 (except for Chao), suggesting that the treatments were significantly different. All indices are higher in 5% than 10% fermented Romaine lettuce brine. Chao value was not significantly different between the two samples. This means that the diversity of both samples were the same. The diversity was estimated from abundance of individuals belonging to a certain taxa. The dissimilarity (Whittaker β-diversity index) between bacterial communities of the two samples was 0.34831. This indicates that there were differences in composition of lower taxa between the samples as supported by the phylogenetic tree (data not shown).

**Table 2:** Lower Taxa Diversity indices.

Brine concentration	Simpson (1-D)	Shannon (H′)	True diversity	Evenness	Equitability	Margalef Richness	Chao
10%	0,5917±0,002	1,169±0,007	3,219±0,0227	0,083±0,0006	0,319±0,002	3,305±0.00	39,6±5,222
5%	0,7185±0,002	1,725±0,008	5,613±0,045	0,117±0,001	0,4457±0,002	4,097±0.00	48±3,464
One-way ANOVA p-value <0.05	Significantly different	Significantly different	Significantly different	Significantly different	Significantly different	Significantly different	Not significantly different

To the best of our knowledge, this is the first metagenomic profiling of bacterial community structure in different concentration of Romaine lettuce fermented brine. The data shows that the growth of most of lactic acid bacteria (LAB) (such as *Lactococcus*, *Leuconostoc*, and some of *Bacillus* and Enterobacteriaceae) which are beneficial for vegetable preservation as well as health was suppresed in 10% brine. This data will be useful in optimization of brine concentration for safety and quality control of vegetable fermentation.

## 2 Experimental design, materials, and methods

### 2.1 Sample preparation

Romaine lettuce was used in this experiment. The vegetables were obtained from local hydroponic farm. The leaves were cut out from the stem and put in a fermentation bottle. Ballast glasses were placed on the leaves to prevent them from floating. In each bottle, 5% and 10% brine solution were poured, respectively, to allow the vegetables fully immersed in the brine. Fermentation process was conducted for 4 days in the dark chamber at room temperature (28–30 °C). After 4 days, the fermented brines were used for metagenomic analysis.

### 2.2 DNA extraction and metagenome sequencing

The gDNA was extracted using ZymoBiomics DNA Mini Kit (Zymo Research) according to protocol from the manufacturer. The hypervariable V3–V4 regions of 16S rRNA gene were amplified using MyTaq™ HS Red Mix (Bioline, BIO-25044) in Agilent SureCycler 8800 Thermal Cycler. The quality and quantity of the genomic DNA were evaluated by electrophoresis on a 0.8% agarose gel followed by staining with GelRedTM (Biotium, CA, USA) and analysed using NanoDrop 1000 (Thermo Scientific, Wilmington, DE, USA). The amplicon library was prepared using an Illumina two-step PCR protocol. The first stage was aimed to generate PCR products that targeted V3–V4 regions using Nextera-style tag sequences (overhang sequences). The second stage used sample specific Illumina Nextera XT dual indices (Nextera XT i7 index and Nextera XT i5 index). Assessment of the quality and quantity of the final library products was conducted using TapeStation 4200 from Agilent Technologies, HelixyteTM green ddsDNA quantifying reagent and qPCR using Jetseq library quantification Lo-Rox kit from Bioline (London, UK). Following the protocol from the Illumina, the paired-end sequences were generated in 2 × 300bp format from MiSeq.

### 2.3 Bioinformatics analysis

The sequence adapters of the raw sequences were removed using Bbmap and merged using BBMerge (BBTools package). The sequences were aligned, trimmed, and chimeras were removed. All reads were clustered into OTU using UCLUST (de novo) at 97% similarity. Singleton and doubletons were removed prior to taxonomy and diversity analysis. The samples then rarefied to the lowest number of reads among all samples. The Greengene Database [3] was used based on RDP Classifier [4] algorithm to annotate taxonomic information for each representative sequence. Because more than one OTU can be assigned to the same species, the OTUs were then combined together for further analysis.

### 2.4 Bacterial community structure analysis

The α- and β-indices were calculated using PAST3 software [5].
